# 4-Hydr­oxy-5-(4-methoxy­phen­yl)pyrrolidin-2-one

**DOI:** 10.1107/S1600536808003899

**Published:** 2008-02-13

**Authors:** M. Fazli Mohammat, Zurina Shaameri, A. Sazali Hamzah, Hoong-Kun Fun, Suchada Chantrapromma

**Affiliations:** a Institute of Science, Universiti Teknologi MARA, 40450 Shah Alam, Selangor, Malaysia; b X-ray Crystallography Unit, School of Physics, Universiti Sains Malaysia, 11800 USM, Penang, Malaysia; c Department of Chemistry, Faculty of Science, Prince of Songkla University, Hat-Yai, Songkhla 90112, Thailand

## Abstract

In the title compound, C_11_H_13_NO_3_, the pyrrolidin-2-one ring is in an envelope conformation with the hydroxyl and 4-methoxy­phenyl substituents mutually *cis*. The methoxy group is slighty twisted away from the mean plane of the attached benzene ring. The mol­ecules are arranged into screw chains along the *c* axis. These chains are inter­connected *via* inter­molecular O—H⋯O and N—H⋯O hydrogen bonds into sheets parallel to the *ac* plane. The crystal structure is further stabilized by weak inter­molecular C—H⋯O and C—H⋯π inter­actions.

## Related literature

For details of ring conformations, see: Cremer & Pople (1975 ▶). For the biological properties of pyrrolidine alkaloids, see for example: Iida *et al.* (1986 ▶); Royles (1996 ▶). For the syntheses of compounds containing the tetra­mic acid ring, see for example: Chandrasekhar *et al.* (2006 ▶); Gurjar *et al.* (2006 ▶); Yoda *et al.* (1996 ▶). For bond-length data, see: Allen *et al.* (1987 ▶).
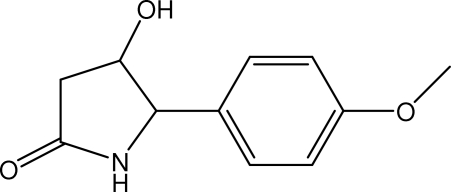


## Experimental

### 

#### Crystal data

C_11_H_13_NO_3_
*M* *_r_* = 207.22
Orthorhombic, 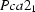

*a* = 11.9862 (6) Å
*b* = 11.6251 (6) Å
*c* = 7.1539 (4) Å
*V* = 996.83 (9) Å^3^
*Z* = 4
Mo *K*α radiation
μ = 0.10 mm^−1^
*T* = 100.0 (1) K
0.43 × 0.20 × 0.17 mm

#### Data collection

Bruker SMART APEX2 CCD area-detector diffractometer
Absorption correction: multi-scan (*SADABS*; Bruker, 2005 ▶) *T*_min_ = 0.958, *T*_max_ = 0.983
8681 measured reflections
1562 independent reflections
1218 reflections with *I* > 2σ(*I*)
*R*_int_ = 0.066

#### Refinement

*R*[*F* ^2^ > 2σ(*F* ^2^)] = 0.047
*wR*(*F* ^2^) = 0.109
*S* = 1.09
1562 reflections
145 parameters
1 restraint
H atoms treated by a mixture of independent and constrained refinement
Δρ_max_ = 0.22 e Å^−3^
Δρ_min_ = −0.24 e Å^−3^

### 

Data collection: *APEX2* (Bruker, 2005 ▶); cell refinement: *APEX2*; data reduction: *SAINT* (Bruker, 2005 ▶); program(s) used to solve structure: *SHELXTL* (Sheldrick, 2008 ▶); program(s) used to refine structure: *SHELXTL*; molecular graphics: *SHELXTL*; software used to prepare material for publication: *SHELXTL* and *PLATON* (Spek, 2003 ▶).

## Supplementary Material

Crystal structure: contains datablocks global, I. DOI: 10.1107/S1600536808003899/sj2463sup1.cif

Structure factors: contains datablocks I. DOI: 10.1107/S1600536808003899/sj2463Isup2.hkl

Additional supplementary materials:  crystallographic information; 3D view; checkCIF report

## Figures and Tables

**Table 1:** Hydrogen-bond geometry (Å, °) *Cg*1 is the centroid of the C5–C10 ring.

*D*—H⋯*A*	*D*—H	H⋯*A*	*D*⋯*A*	*D*—H⋯*A*
N1—H1*N*1⋯O2^i^	0.88 (4)	2.05 (4)	2.917 (3)	167 (4)
O2—H1*O*2⋯O3^ii^	0.90 (4)	1.98 (4)	2.800 (2)	152 (3)
C3—H3*A*⋯O1^iii^	0.98	2.33	3.193 (3)	146
C11—H11*A*⋯O1^iv^	0.96	2.49	3.395 (3)	158
C6—H6*A*⋯*Cg*1^v^	0.93	2.81	3.514 (3)	133
C9—H9*A*⋯*Cg*1^vi^	0.93	2.68	3.554 (3)	157

Symmetry codes: (i) 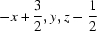; (ii) 
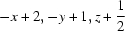
; (iii) 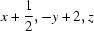; (iv) 
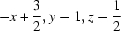
; (v) 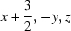; (vi) 
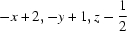
.

**Fig. 1.**
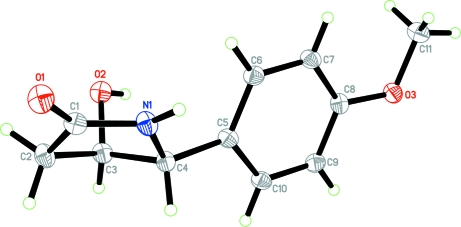
The molecular structure of (I), showing 40% probability displacement ellipsoids and the atomic numbering.

**Fig. 2.**
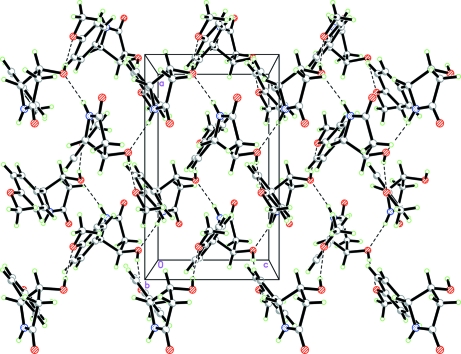
The crystal packing of (I), viewed along the b axis. Hydrogen bonds were drawn as dashed lines.

## supplementary crystallographic information

### Comment

Many naturally occurring compounds containing a tetramic acid ring system such as radicamine, fuligorobin and codonopsinine possess potent antibiotic, antiviral, antifungal, cytotoxic (Royles, 1996) as well as hypotensive activities (Iida *et al.*, 1986). The title compound, C_11_H_13_NO_3_, can act as an essential intermediate in the synthesis of such tetramic acid derivatives (Chandrasekhar *et al.*, 2006; Gurjar *et al.*, 2006; Yoda *et al.*, 1996), which eventually can be used as a template in multi-step syntheses of biologically active natural products. We have synthesized the title compound (I) and its structure is reported here, Fig. 1.

In (I), the pyrrolidine-2-one ring adopts an envelope conformation with atom C3 displaced from the C1/C2/C3/N1 plane by 0.219 (3) Å, and with puckering parameters (Cremer & Pople, 1975) Q = 0.357 (3) Å and φ = 117.9 (4)°. The bond angles around C1 atom are indicative of *sp*^2^ hybridization. The hydroxyl and 4-methoxyphenyl substituents are attached to the pyrrolidin-2-one ring at atom C3 and C4, respectively and is in *cis*-configuration (Fig. 1). The methoxy group is slightly twisted away from the mean plane of the phenyl ring as shown by the torsion angle C11–O3–C8–C7 = -5.2 (4)° All bond lengths and angles show normal values (Allen *et al.*, 1987)

In the crystal packing of the title compound (Fig. 2), the molecules are arranged into screw chains along the *c* direction. These chains are interconnected *via* intermolecular O—H···O and N—H···O hydrogen bonds (Table 1) into sheets parallel to the *ac* plane. The crystal is further stabilized by weak intermolecular C—H···O and C—H···π interactions; C6—H6A···*Cg*_1_ (symmetry code: 3/2 - *x*, *y*, 1/2 + *z*) and C9—H9A··· *Cg*_1_ (symmetry code: 2 - *x*, 1 - *y*, -1/2 + *z*), *Cg*_1_ is the centroid of C5–C10 phenyl ring.

### Experimental

The synthetic approach to the title compound began with the esterification of *p*-hydroxyphenylglycine (10.00 g, 60.10 mmol) and thionyl chloride in methanol to give the ester product (10.30 g, 95%). Amine protection (10.00 g, 54.9 mmol) was then carried out using *tert*-butoxycarbonyl (Boc_2_O) and triethylamine (Et_3_N) in tetrahydrofuran (THF) to give the *N*-Boc protected product in 85% yield (13.12 g). The hydroxyl functional group (13.01 g, 46.66 mmol) was protected by converting it to the methyl ether using potassium carbonate and methyl iodide (12.72 g, 93%). Condensation between the *N*-Boc methyl ester (8.30 g, 28.30 mmol) and methyl malonyl chloride in equimolar amounts furnished an intermediate diester (10.60 g, 95%). Dieckmann cyclization of this intermediate diester (5.50 g, 13.99 mmol) with potassium *tert*-butoxide (*t*-BuOK) in toluene gave the carbon skeleton β,β diketoester in 45% yield (1.65 g). Demethoxycarbonylation of the β,β diketoester (0.30 g, 1.1 mmol) was successfully carried out by refluxing in 50 ml acetonitrile to give the basic pyrrolidinone ring skeleton (0.23 g, 99%). Reduction of this diketone (0.16 g, 0.77 mmol) was then carried out in sodium borohydride/methanol at 273 K to give the title compound (0.04 g, 24%). Single crystals suitable for *X*-ray structure determination were obtained by slow evaporation of an ethyl acetate-petroleum ether (2:1 *v*/*v*) solution after several days.

### Refinement

H atoms attached to O and N atoms were located in a difference Fourier map and were refined isotropically. H atoms bound to C were placed in calculated positions with d(C—H) = 0.93 Å, *U*_iso_=1.2*U*_eq_ (C) for aromatic 0.98 Å, *U*_iso_ = 1.2*U*_eq_ (C) for CH, 0.97 Å, *U*_iso_ = 1.2*U*_eq_ (C) for CH_2_, 0.96 Å, *U*_iso_ = 1.5*U*_eq_ (C) for CH_3_ atoms. A rotating group model was used for the methyl groups. A total of 1121 Friedel pairs were merged before final refinement as there is no large anomalous dispersion for the determination of the absolute configuration.

### Crystal data

**Table:**

C_11_H_13_NO_3_	*F*_000_ = 440
*M**_r_* = 207.22	*D*_x_ = 1.381 Mg m^−^^3^
Orthorhombic, *P**c**a*2_1_	Mo *K*α radiation λ = 0.71073 Å
Hall symbol: P 2c -2ac	Cell parameters from 1562 reflections
*a* = 11.9862 (6) Å	θ = 1.8–30.0º
*b* = 11.6251 (6) Å	µ = 0.10 mm^−^^1^
*c* = 7.1539 (4) Å	*T* = 100.0 (1) K
*V* = 996.83 (9) Å^3^	Block, colorless
*Z* = 4	0.43 × 0.20 × 0.17 mm

### Data collection

**Table:**

Bruker SMART APEX2 CCD area-detector diffractometer	1562 independent reflections
Radiation source: fine-focus sealed tube	1218 reflections with *I* > 2σ(*I*)
Monochromator: graphite	*R*_int_ = 0.066
Detector resolution: 8.33 pixels mm^-1^	θ_max_ = 30.0º
*T* = 100.0(1) K	θ_min_ = 1.8º
ω scans	*h* = −16→13
Absorption correction: multi-scan(SADABS; Bruker, 2005)	*k* = −16→16
*T*_min_ = 0.958, *T*_max_ = 0.983	*l* = −10→9
8681 measured reflections	

### Refinement

**Table:**

Refinement on *F*^2^	Secondary atom site location: difference Fourier map
Least-squares matrix: full	Hydrogen site location: inferred from neighbouring sites
*R*[*F*^2^ > 2σ(*F*^2^)] = 0.047	H atoms treated by a mixture of independent and constrained refinement
*wR*(*F*^2^) = 0.109	*w* = 1/[σ^2^(*F*_o_^2^) + (0.0466*P*)^2^ + 0.0367*P*] where *P* = (*F*_o_^2^ + 2*F*_c_^2^)/3
*S* = 1.09	(Δ/σ)_max_ < 0.001
1562 reflections	Δρ_max_ = 0.22 e Å^−^^3^
145 parameters	Δρ_min_ = −0.24 e Å^−^^3^
1 restraint	Extinction correction: none
Primary atom site location: structure-invariant direct methods	

### Special details

**Table:**

Experimental. The data was collected with the Oxford Cyrosystem Cobra low-temperature attachment.
Geometry. All e.s.d.'s (except the e.s.d. in the dihedral angle between two l.s. planes) are estimated using the full covariance matrix. The cell e.s.d.'s are taken into account individually in the estimation of e.s.d.'s in distances, angles and torsion angles; correlations between e.s.d.'s in cell parameters are only used when they are defined by crystal symmetry. An approximate (isotropic) treatment of cell e.s.d.'s is used for estimating e.s.d.'s involving l.s. planes.
Refinement. Refinement of *F*^2^ against ALL reflections. The weighted *R*-factor *wR* and goodness of fit *S* are based on *F*^2^, conventional *R*-factors *R* are based on *F*, with *F* set to zero for negative *F*^2^. The threshold expression of *F*^2^ > σ(*F*^2^) is used only for calculating *R*-factors(gt) *etc*. and is not relevant to the choice of reflections for refinement. *R*-factors based on *F*^2^ are statistically about twice as large as those based on *F*, and *R*- factors based on ALL data will be even larger.

### Fractional atomic coordinates and isotropic or equivalent isotropic displacement parameters (Å^2^)

**Table:**

	*x*	*y*	*z*	*U*_iso_*/*U*_eq_	
O1	0.69354 (13)	1.02873 (16)	1.1878 (3)	0.0282 (5)	
O2	0.93282 (14)	0.80442 (16)	1.3464 (3)	0.0238 (4)	
H1O2	0.977 (3)	0.742 (3)	1.345 (6)	0.045 (10)*	
O3	0.87847 (13)	0.33500 (15)	0.8900 (3)	0.0239 (4)	
N1	0.76602 (18)	0.86418 (18)	1.0604 (3)	0.0228 (5)	
H1N1	0.704 (3)	0.838 (3)	1.009 (6)	0.043 (10)*	
C1	0.7727 (2)	0.9651 (2)	1.1524 (4)	0.0223 (6)	
C2	0.89395 (19)	0.9844 (2)	1.2029 (5)	0.0243 (6)	
H2A	0.9012	1.0105	1.3311	0.029*	
H2B	0.9279	1.0408	1.1206	0.029*	
C3	0.94736 (19)	0.8665 (2)	1.1771 (4)	0.0222 (6)	
H3A	1.0262	0.8725	1.1423	0.027*	
C4	0.8761 (2)	0.8158 (2)	1.0169 (4)	0.0207 (6)	
H4A	0.9018	0.8495	0.8989	0.025*	
C5	0.8762 (2)	0.6876 (2)	0.9962 (4)	0.0199 (6)	
C6	0.79693 (19)	0.6171 (2)	1.0788 (4)	0.0221 (6)	
H6A	0.7435	0.6497	1.1568	0.027*	
C7	0.79523 (19)	0.4993 (2)	1.0483 (4)	0.0229 (6)	
H7A	0.7403	0.4538	1.1030	0.028*	
C8	0.8756 (2)	0.4503 (2)	0.9363 (4)	0.0214 (6)	
C9	0.95804 (18)	0.5175 (2)	0.8550 (4)	0.0226 (6)	
H9A	1.0130	0.4839	0.7812	0.027*	
C10	0.95734 (19)	0.6345 (2)	0.8850 (4)	0.0220 (6)	
H10A	1.0124	0.6796	0.8298	0.026*	
C11	0.7980 (2)	0.2604 (2)	0.9764 (5)	0.0287 (7)	
H11A	0.8057	0.1841	0.9266	0.043*	
H11B	0.7242	0.2885	0.9512	0.043*	
H11C	0.8103	0.2588	1.1089	0.043*	

### Atomic displacement parameters (Å^2^)

**Table:**

	*U*^11^	*U*^22^	*U*^33^	*U*^12^	*U*^13^	*U*^23^
O1	0.0208 (9)	0.0270 (10)	0.0367 (12)	0.0068 (8)	−0.0016 (9)	−0.0014 (9)
O2	0.0184 (8)	0.0253 (10)	0.0276 (11)	0.0048 (8)	−0.0014 (8)	0.0003 (9)
O3	0.0211 (8)	0.0217 (9)	0.0288 (11)	0.0006 (7)	0.0017 (8)	−0.0027 (8)
N1	0.0133 (10)	0.0230 (11)	0.0321 (14)	0.0000 (9)	−0.0023 (10)	−0.0027 (10)
C1	0.0202 (12)	0.0228 (12)	0.0238 (16)	0.0016 (10)	−0.0008 (10)	0.0026 (12)
C2	0.0194 (12)	0.0219 (13)	0.0317 (16)	−0.0007 (10)	−0.0029 (11)	−0.0019 (12)
C3	0.0137 (11)	0.0240 (13)	0.0289 (14)	0.0009 (10)	0.0009 (11)	−0.0020 (12)
C4	0.0179 (12)	0.0200 (13)	0.0242 (14)	0.0006 (10)	−0.0001 (10)	−0.0017 (11)
C5	0.0137 (11)	0.0218 (13)	0.0243 (15)	0.0006 (10)	0.0000 (10)	0.0009 (11)
C6	0.0170 (11)	0.0255 (13)	0.0239 (14)	0.0016 (9)	0.0024 (11)	−0.0013 (12)
C7	0.0167 (12)	0.0243 (13)	0.0278 (15)	−0.0008 (10)	0.0027 (11)	0.0001 (12)
C8	0.0178 (12)	0.0212 (13)	0.0251 (15)	0.0021 (10)	−0.0030 (10)	−0.0015 (11)
C9	0.0173 (12)	0.0254 (13)	0.0252 (14)	0.0029 (9)	0.0040 (11)	−0.0021 (12)
C10	0.0170 (12)	0.0239 (13)	0.0251 (14)	−0.0011 (9)	0.0024 (11)	0.0013 (13)
C11	0.0250 (13)	0.0263 (15)	0.0346 (18)	−0.0003 (11)	0.0035 (12)	0.0030 (14)

### Geometric parameters (Å, °)

**Table:**

O1—C1	1.229 (3)	C4—H4A	0.9800
O2—C3	1.420 (3)	C5—C6	1.387 (4)
O2—H1O2	0.90 (3)	C5—C10	1.399 (4)
O3—C8	1.381 (3)	C6—C7	1.387 (4)
O3—C11	1.437 (3)	C6—H6A	0.9300
N1—C1	1.347 (3)	C7—C8	1.376 (4)
N1—C4	1.468 (3)	C7—H7A	0.9300
N1—H1N1	0.88 (4)	C8—C9	1.387 (3)
C1—C2	1.515 (3)	C9—C10	1.378 (4)
C2—C3	1.524 (4)	C9—H9A	0.9300
C2—H2A	0.9700	C10—H10A	0.9300
C2—H2B	0.9700	C11—H11A	0.9600
C3—C4	1.547 (4)	C11—H11B	0.9600
C3—H3A	0.9800	C11—H11C	0.9600
C4—C5	1.498 (3)		
			
C3—O2—H1O2	109 (3)	C3—C4—H4A	108.2
C8—O3—C11	117.8 (2)	C6—C5—C10	117.2 (2)
C1—N1—C4	112.6 (2)	C6—C5—C4	123.0 (2)
C1—N1—H1N1	123 (2)	C10—C5—C4	119.7 (2)
C4—N1—H1N1	123 (2)	C7—C6—C5	121.8 (2)
O1—C1—N1	125.4 (2)	C7—C6—H6A	119.1
O1—C1—C2	127.0 (2)	C5—C6—H6A	119.1
N1—C1—C2	107.6 (2)	C8—C7—C6	119.3 (2)
C1—C2—C3	103.9 (2)	C8—C7—H7A	120.3
C1—C2—H2A	111.0	C6—C7—H7A	120.3
C3—C2—H2A	111.0	C7—C8—O3	124.0 (2)
C1—C2—H2B	111.0	C7—C8—C9	120.7 (2)
C3—C2—H2B	111.0	O3—C8—C9	115.3 (2)
H2A—C2—H2B	109.0	C10—C9—C8	119.1 (2)
O2—C3—C2	107.6 (2)	C10—C9—H9A	120.5
O2—C3—C4	111.8 (2)	C8—C9—H9A	120.5
C2—C3—C4	101.6 (2)	C9—C10—C5	121.9 (2)
O2—C3—H3A	111.8	C9—C10—H10A	119.1
C2—C3—H3A	111.8	C5—C10—H10A	119.1
C4—C3—H3A	111.8	O3—C11—H11A	109.5
N1—C4—C5	113.8 (2)	O3—C11—H11B	109.5
N1—C4—C3	101.1 (2)	H11A—C11—H11B	109.5
C5—C4—C3	116.9 (2)	O3—C11—H11C	109.5
N1—C4—H4A	108.2	H11A—C11—H11C	109.5
C5—C4—H4A	108.2	H11B—C11—H11C	109.5
			
C4—N1—C1—O1	172.0 (3)	N1—C4—C5—C10	154.0 (2)
C4—N1—C1—C2	−8.0 (3)	C3—C4—C5—C10	−88.6 (3)
O1—C1—C2—C3	164.1 (3)	C10—C5—C6—C7	−2.1 (4)
N1—C1—C2—C3	−15.8 (3)	C4—C5—C6—C7	176.1 (3)
C1—C2—C3—O2	−86.1 (3)	C5—C6—C7—C8	1.4 (4)
C1—C2—C3—C4	31.4 (3)	C6—C7—C8—O3	−176.9 (2)
C1—N1—C4—C5	154.0 (2)	C6—C7—C8—C9	0.3 (4)
C1—N1—C4—C3	27.9 (3)	C11—O3—C8—C7	−5.2 (4)
O2—C3—C4—N1	79.4 (2)	C11—O3—C8—C9	177.4 (2)
C2—C3—C4—N1	−35.1 (2)	C7—C8—C9—C10	−1.2 (4)
O2—C3—C4—C5	−44.7 (3)	O3—C8—C9—C10	176.3 (2)
C2—C3—C4—C5	−159.2 (2)	C8—C9—C10—C5	0.4 (4)
N1—C4—C5—C6	−24.2 (4)	C6—C5—C10—C9	1.2 (4)
C3—C4—C5—C6	93.2 (3)	C4—C5—C10—C9	−177.1 (2)

### Hydrogen-bond geometry (Å, °)

**Table:**

*D*—H···*A*	*D*—H	H···*A*	*D*···*A*	*D*—H···*A*
N1—H1N1···O2^i^	0.88 (4)	2.05 (4)	2.917 (3)	167 (4)
O2—H1O2···O3^ii^	0.90 (4)	1.98 (4)	2.800 (2)	152 (3)
C3—H3A···O1^iii^	0.98	2.33	3.193 (3)	146
C11—H11A···O1^iv^	0.96	2.49	3.395 (3)	158
C6—H6A···Cg1^v^	0.93	2.81	3.514 (3)	133
C9—H9A···Cg1^vi^	0.93	2.68	3.554 (3)	157

Symmetry codes: (i) −*x*+3/2, *y*, *z*−1/2; (ii) −*x*+2, −*y*+1, *z*+1/2; (iii) *x*+1/2, −*y*+2, *z*; (iv) −*x*+3/2, *y*−1, *z*−1/2; (v) *x*+3/2, −*y*, *z*; (vi) −*x*+2, −*y*+1, *z*−1/2.
